# In Memoriam

**Published:** 1888-06

**Authors:** 


### Editorial.

### IN MEMORIAM.

### Augustus Reginald Davidson, M. D.,

Died May 25, 1888, aged forty-three years.

This number of the Journal conveys to our readers a heavy sorrow in the announcement of the death of our esteemed collaborator, Dr. A. R. Davidson, who for many years has been associated in its editorial management. Dr. Davidson fell a victim to adynamic pneumonia, after a very brief illness. The contents of this number were arranged by his hands, and on his sick-bed he gave directions as to original communications, and entrusted to Dr. Campbell, the associate editor, the work of making the selections. So sudden was the fatal termination of the disease that, among the profession in the city, many were taken by surprise when the sad event was announced in the secular press.

Dr. Davidson was born in the province of Ontario, April 11, 1845, and was educated chiefly under the direct personal supervision of his father, who was a highly-educated clergyman of the Anglican church. Through the influence of his brother, his early life was devoted to the drug business, which gave direction to his future course, in affording him the opportunity to cultivate his taste for chemistry, for which he early manifested an aptitude. In 1868, he attended a course of medical lectures at the University of Michigan, with a view to the profession of medicine, to which his attention was directed, through the failure of commercial life, to satisfy his scientific and intellectual tastes. Circumstances interfering with the immediate consummation of this plan, he came to Buffalo in 1869 and assumed the management of Peabody's drug store, at that time the most extensive establishment in this city, a position he filled with great ability, securing, in a larger measure than is usual, the confidence of the profession, in the intelligence and experience he brought to his duties. For a brief period, he afterwards engaged in the drug busines on his own responsibility, at the corner of Main and Chippewa streets, which gave him the means to complete his medical course at the University of Buffalo, from which he took the degree of Doctor of Medicine in 1878. During the year or two previous to his graduation, he assisted Professor Charles A. Doremus in the course of chemistry, delivered by that accomplished teacher, in the college. In 1878, with Drs. Howe, Van Peyma and Mynter, and the writer, the BUFFALO MEDICAL AND SURGICAL JOURNAL was purchased of the late Dr. Julius F. Miner, and Dr. Davidson assumed the business management, and aided materially in the editorial work. On the resignation of Dr. E. V. Stoddard, of the chair of Materia Medica in the Buffalo Medical College, in 1881, Dr. Davidson was requested, by the late Professor James P. White, to accept the position of lecturer, and he gave, in the session of 1881 and 1882, a course of lectures on materia medica and therapeutics, for which his extensive, practical knowledge of the subject and scientific tastes peculiarly fitted him. In 1882 and 1883, he passed several months in Philadelphia, taking the post-graduate course, at the completion of which he went to London, and specially studied renal and skin diseases, with a view to engage in this specialty in his future practice. At the organization of the Medical Department of Niagara University in 1883, he accepted the Professorship of Medical Chemistry, Toxicology and Dermatology, which he held at the time of his death, and the trustees of the University, in acknowledgment of his fidelity to the high position to which he was called, conferred upon him, in 1886, the degree, "*Ad eundum Doctoris in Medicina.*"

With this brief outline of his personal history and his life, it will be justly inferred that Dr. Davidson depended upon his own energies and ability for his success. He possessed an unusual aptitude for scientific investigations and research. He was an accomplished chemist, and his accurate and skillful analytical work gave him the leading position as a chemist in this section of the Empire State. It is to his indefatigable labors and industry that every step in his onward progress was due. He was strong in his principles and in the advocacy of them. He was just and honorable in all his dealings. Indeed, it may be said that he was at times misunderstood in the attitude he assumed on many important questions which have divided the profession in this city, on account of his conscientious purpose to be *just* in his position towards antagonizing elements.

Dr. Davidson was strong in his religious convictions and principles. He accepted the teachings of the Catholic party in the American church, and his daily life exemplified the faith he embraced. His honesty and consistency were carried into all his public and private duties. The confidence of the profession and the community in which he lived, were attested at the solemn services at his funeral, and in the expressions of sympathy which his too early death called forth.

It may be said in truth, that this life which promised so much for him in a field of professional labor peculiarly adapted to his tastes and acquirements, was cut short by overwork. He was unceasing in his labors and studies, and gave himself but little of needed rest and recreation. The loss sustained by his inopportune death seems to us irreparable. The void is difficult to fill in the profession, in the college in which he was a most successful teacher, and in the community, of which he was an honored and respected citizen. To his associates on the JOURNAL, he was endeared as only a just and noble man could be esteemed, and we part with him with loving memories of past associations, with deep appreciation of his moral, intellectual and professional worth, and with devout thankfulness that the profession of medicine should be chosen by a man so pure and just, so noble in his aspirations, and earnest and honorable in his life-work, as the congenial field in which to consecrate the superior gifts with which he was endowed.

We append the action of the Faculty of the Medical Department of Niagara University, and of the County Medical Society, which truthfully portrays the high estimation in which he was held by those who knew him most intimately:

Entered into rest May 25, 1888. Augustus Reginald Davidson, M. D., Professor of Medical Chemistry and Toxicology in the Medical Department of Niagara University.

Death has entered again our faculty and removed one of the most beloved of our members. An earnest student, an apt and successful teacher, a physician endowed with fine intellectual gifts and broad and thorough medical attainments, Prof. Davidson had already risen to a position of influence in his profession, and commanded in a rare degree the confidence and respect of the community. It is acknowledged that in his special work he was unequaled, and his judgment and skill were sought by his brother physicians. He was strong in his convictions of right, and possessed the courage of expressing and adhering to the high principles which he had established as the rule of his life. Few men reach at his age the high position in the profession which his energies and ability had already commanded. The loss of such a man is felt beyond the circle of friends and acquaintances, for the world recognizes noble gifts and conscientious devotion to duty such as were exemplified in the life now brought to its close.

We desire to express to the family of our deceased associate the deepness of sorrow we feel at the loss of a noble son and brother, a faithful husband and a loving father. We join in their bereavement, for the loss is not theirs alone; it is ours. We feel that the deep devotion to religious principles and duty which characterized his life, brings the reward promised to faithful stewardship. While humbly submitting to the divine will which takes from our work a brother beloved, and a teacher eminent and successful, a citizen of honor and uprightness, a physician of rare attainments and skill, we express our thankfulness for the example of such a life.

The Erie County Medical Society, at a meeting held May 26, 1888, adopted the following memorial:

*IN MEMORIAM.*

Augustus Reginald Davidson, M. D.

Died May 25, 1888, aged 43 years.

The medical profession of this city and county are summoned again to recognize the strong arm of death, which has suddenly taken from us a Christian gentleman, an honored citizen and an accomplished physician. We desire to bear testimony to the assiduous labors and steadfastness of purpose which has made his life an example worthy of imitation. He was endowed with superior natural gifts, and with high and noble aspirations, in the profession to which his maturer years were devoted. He had consecrated every effort of mind and body for the high office of a physician, and while he had not yet attained the meridian of life, he had reached an eminence in professional circles which were the deserved reward of his industry and labors.

This society, of which Dr. Davidson was an honored member, desires to place on record its high appreciation of his moral and intellectual worth, his superior scientific and professional attainments, and with pride to note the rapid progress he made towards the goal of his ambition, a life of usefulness towards his fellow-man. We desire to bear our testimony to his sound judgment as a diagnostician, to the broad professional culture which made him an exceptionally accurate therapeutist, and contributed to his success in the special department of medical science to which he devoted his energies. We also recognize in his life a rigid adherence to principle and to duty, and an earnest conviction that the medical profession claimed from him the consecration of every power he possessed of mind and heart for the noble office of a "healer of the sick."

We beg the precious privilege to share with the family of our deceased associate in the sorrow this untimely death has brought to them. Their loss is greater than any words of ours can express, and we pray that our sympathy may assuage in a measure their grief. While bowing to the divine will which takes from the scenes of active life an honored physician, a devoted husband and father, we have reason for thankfulness that our profession was the chosen field, and furnished the incentive for the noble qualities of head and heart which his life so beautifully exemplified.

